# Secondary ALL after Successful Treatment of Ewing's Sarcoma: A Case Report

**Published:** 2016-10-01

**Authors:** Kourosh Goudarzi Pour, Samin Alavi, Shahin Shamsian, Roxana Aghakhani, Mohammad Taghi Arzanian, Hesameddin Hoseini Tavassol, Zahra Eydian, Reyhaneh Kazemi

**Affiliations:** Pediatric Congenital Hematologic Disorders Research Center, Shahid Beheshti University of Medical Sciences, Tehran, Iran

**Keywords:** Chemoradiotherapy, Leukemia, Ewing sarcoma

## Abstract

Treatment with intensification of chemotherapy using alkylating agents and Topoisomerase II inhibitors and radiotherapy has improved the outcome of patients with solid tumors such as Ewing’s sarcoma. However, there are several reports of secondary malignancy following treatment of these tumors. In this article, we describe a 12 years old girl with ALL who had Ewing’s sarcoma when she was 8 years old and underwent successful treatment but after two and half years at 12 years old, she came back with pallor and muscular pain.

## Introduction

 The second most common malignant primary bone tumor is Ewing’s sarcoma. It occurs mostly in children. Despite the advances in its treatment with chemotherapy and radiotherapy, the treatment is accompanied with complications such as secondary malignancies. Prevalence of these secondary malignancies differs in reports.

Several large cohort studies with long follow-up times of childhood cancer survivors have reported three to six fold increased risk of a second cancer, in comparison with the general population and beside 5–10% of children treated for first malignancy have developed subsequent second tumors.^1^^,^^2^ Also, the role of radiotherapy and some anticancer drug in developing secondary malignancy is obvious. Although the overwhelming number of cases of secondary acute leukemia (AL) is classified as AML, a small percentage of cases are acute lymphoblastic leukemia (ALL).^3^^,^^4^ Studies have shown that the relative risk of secondary leukemia raises with increasing doses of either radiation or epipodophyllotoxins and alkylating agents.^5^^-^^6^

This article reports an 8 years old girl diagnosed with Pre B cell leukemia following chemotherapy and radiotherapy for treatment of Ewing's sarcoma. Informed consent was obtained of her parents for the study.

## CASE PRESENTATION

 An eight year-old girl was admitted in our center with lower extremity dull bone pain. Imaging studies were performed and MRI showed a lesion in distal metaphysial area of knee which had involved soft tissue as well. Pathologic study revealed blue small round cell tumor and immunohistochemistry staining for CD99 (MIC2) was positive but cytogenetic study for t (11, 22) was negative. CT scans of chest, abdomen and pelvis were unremarkable. In her Initial paraclinical studies, CBC was normal and she had ESR=114, alkaline phosphatase=245 and LDH=360. Also, bone marrow aspiration and biopsy were unremarkable. The patient underwent chemotherapy and radiotherapy according to the diagnosis of Ewing’s sarcoma. After twenty months of follow-up, MRI showed remarkable improvement and the treatment was ceased.

In 2014, four years after the primary diagnosis she was admitted again with severe pallor and weakness. She had cervical and axillary’s lymphadenopathies with obvious splenomegaly. In her paraclinical studies, she had WBC=208000/dL (PMN=2% Lymphocytes=26% Blast=63%), Hb=4.7 mg/dl Plt=105000/dl and ESR=107. Bone marrow aspiration and biopsy were performed and showed acute lymphoblastic leukemia and the results of flowcytometry studies (CD10=0.1%, CD19=78%, CD20=1.9%, HLA-DR=66%) and cytogenetic study which was positive for t (4;11) were compatible with pre B cell leukemia. The patient underwent chemotherapy and now she is in remission phase.

## Discussion

 The treatment of pediatric cancer has been improved in recent years with aggressive use of multimodal therapy, yielding an overall 5-year survival of 78%. This has brought forward the emerging spectrum of long-term sequel among cancer survivors, of which the most life threatening is subsequent second malignancies.^7^ Previous studies have indicated that the cumulative incidence of a second neoplasm is 3.2% at 20 years from the original diagnosis.^8^

Vesudevan et al. in a study among 31685 cases of solid tumor reported overall 177 patients were diagnosed with unique second malignancy before 20 years of age (0.56%) and followed-up for an average of 8.5 years (range 2 month to 30.8 years, mean 8.5 years). The most common solid tumors were central nervous system (CNS) tumors (22.6%), sarcomas (15.8%) and retinoblastoma (14.1%) and bone tumor (13%). The most common second malignancy histologic subtype in children was hematologic tumors (35.5%), most of which were leukemia, followed by malignant bone tumors (18.6%) and sarcomas (14.8%). Approximately, 85% of the hematologic second malignancies were seen within the first 5 years from the diagnosis of primary solid tumor (mean latent period of 3.100 years). In contrast, solid second tumors had a mean latency of 11.6 years. In children both male and female patients had similar incidence of second malignancy.^9^

Kuttesch et al. reported a study among 266 cases with primary Ewing’s sarcoma. After a median follow-up duration of 9.5 years (range, 3.0 to 30), secondary malignancies had been seen in 16 patients including 10 sarcomas (five cases of osteosarcomas, three cases of fibrosarcomas and two cases of malignant fibrous histiocytomas) and six cases of other malignancies had been seen (including acute myeloblastic leukemia, acute lymphoblastic leukemia, meningioma, bronchoalveolar carcinoma, basal cell carcinoma, and cervical carcinoma-in-situ). The second malignancy had been seen in the median latency of 7.6 years following to the diagnosis (3.5 to 25.7 years).^10^

Navid et al. studied a retrospective review of 237 patients with Ewing’s sarcoma from September 1979 to February 2004. Secondary malignancies were found in twelve patients including 8 patients with secondary leukemia (2 ALL, 6 MDS/AML), with median latency of 2.6 years (1.4-19.6 years) subsequent to diagnosis of Ewing’s sarcoma. In four patient's secondary solid tumor had been developed, with a median latency of 8 years (7.4-9.4 years) following the diagnosis of Ewing’s sarcoma.^11^ Overall, constitutional genetic changes play a role in increasing susceptibility to childhood cancer in fewer than 3% of cases.^12^

It is known that radiotherapy for the primary cancer will increase the risk of development of secondary neoplasms in irradiated sites. Alkylating agents and anthracyclines have also been implicated in affecting the radiation therapy-associated risk for secondary malignancies.^13^^,^^14^

## CONCLUSION

 Second malignancies are devastating long-term complications of childhood solid cancer survivors with significant morbidity and mortality. The most common solid primary tumors which are accompanied with increased risk of second malignancies in children are retinoblastoma, CNS tumors and soft tissue sarcomas. The most common one among these secondary malignancies is leukemia. Young children are particularly susceptible to the mutagenic effects of both chemotherapy and radiotherapy, as proven by several studies. Although effective treatment for the initial cancer is principal importance, future therapeutic strategies for childhood cancer should continuously evolve to minimize this potential problem in the long run.^9^
